# Effective deep convolutional neural network with attention mechanism for Alzheimer disease classification

**DOI:** 10.3389/fradi.2025.1698760

**Published:** 2026-01-14

**Authors:** Sathish Kumar Lakshmanan, Maragatharajan Muthusamy, Rajesh Kumar Dhanaraj, Aanjankumar Sureshkumar, Md Shohel Sayeed, Mohamed Yasin Noor Mohamed, Gopal Rathinam

**Affiliations:** 1 School of Computing Science and Engineering, VIT Bhopal University, Bhopal-Indore Highway, Kothrikalan, Sehore, Madhya Pradesh, India; 2 School of Computing, SRM Institute of Science and Technology, Tiruchirappalli, Tamilnadu, India; 3 Symbiosis Institute of Computer Studies and Research (SICSR), Symbiosis International (Deemed University), Pune, India; 4 Centre for Intelligent Cloud Computing, CoE for Advanced Cloud, Faculty of Information Science & Technology, Multimedia University, Jalan Ayer Keroh Lama, Bukit Beruang, Melaka, Malaysia; 5 Faculty of Information Technology, Sultan Qaboos University, Muscat, Oman; 6 Information and Communication Engineering, College of Engineering, University of Buraimi, Al Buraimi, Oman

**Keywords:** Alzheimer's disease, Attention mechanism, Deep convolutional neural networks, Magnetic resonance image, Neuro biomarkers

## Abstract

**Introduction:**

The reports from the Health Organizations indicates a sudden growth in neurocognitive disorders among middle-aged and elderly individuals. The accurate detection of Alzheimer's disease (AD) is essential for improving patient care, specifically during the early stages, where timely risk identification enables individuals to adopt preventive measures before irreversible brain damage occurs. Though, several studies have discovered about computerized approaches for AD, many existing techniques remain limited by inherent methodological constraints and insufficient clinical scrutiny. The current systems struggle to reliably predict the disorder in its initial stages. To reduce the need for frequent clinical visit and lower diagnostic costs, the machine learning and deep learning have emerged as powerful tools for AD detection.

**Methods:**

This work reviews several research relevant on studies on AD and highlights how these computational techniques can support researchers in achieving more efficient and accurate early-stage detection. The Deep Convolutional Neural Network (Deep-CNN) with Attention mechanism is proposed to augment the spatial attention module and multi-class classification of Alzheimer disease stages. The model has trained and evaluated on the OASIS dataset using subject-level which satisfy statistical-validation and standard preprocessing.

**Results:**

The proposed Deep-CNN and attention model focuses the model capacity on diagnostically relevant regions. The proposed model achieved an accuracy of 97%, which is higher than existing methods like SVM with kernels (90.5%), SVM Gaussian radial basis kernel (85%), and traditional CNN (93.5%).

**Discussion:**

The visualizations of attention mechanism are used to increase the interpretability and demonstrate the attention maps which are align with known AD biomarkers. These results indicates that the attention-guided deep models can both improve multi-class MRI classification accuracy and provide clinically useful regional explanations.

## 1 Introduction

The systematic psychiatric research has shown many traditional and non-scientific computational approaches can still yield meaningful insights (1). This interdisciplinary filed contributes to understanding the biomedical processes underlying both healthy brain function and disease conditions. This helps to translate these mechanisms into interpretable medical visualizations. With the rapid global increase in biomedical data (2) and the parallel progress in the machine learning models, the researchers have efficient tools for accurately detecting and staging neurological disorders. These developments fall within the broader scope of computerized dimensional analysis (3, 4). The recent innovations have enabled the integration of multiple patient-centred monitoring systems into clinical decision-making, ultimately improving outcomes for individuals affected by AD. The main aim of these neuroscientific advancements (5, 6) is to enhance early awareness and intervention as timely management is crucial for reducing the risk associated with AD and its progressive cognitive decline. Therefore, the contemporary studies (7, 8) are increasingly focused on building highly efficient machine learning-based frameworks to support AD diagnosis. The predictive models using MRI and CT imaging have shown strong potential in differentiating AD patients from healthy individuals by facilitating early diagnosis. Based on the existing reviews, the study examines comparable research which employs various AD datasets and utilizes MRI, CT, deep learning and machine learning techniques for more efficient disease analysis.

The text manual (9), which is prepared for students and medical practitioners described as a specific group of patients who exhibited severe cell abnormalities that leads to the formation of multiple plaques. These abnormalities involved the loss of nearly 25% of the spinal cortex, which can be replaced by clusters of distinctly coloured neurofibrillary structures. The manual method discussed the most advanced stages of degeneration observed in these cases. During this era, the scientific understanding of AD was still unclear, but Kreapelin summarised the conditions of AD coined the term and characterized as a distinct disorder. The early examination of Auguste Deter's condition (10) was highly uncertain and clear scientific definitions of AD did not emerge until a century later.

In 1998, researches from MG university and MP Institute of neurobiology in Marinated reported that neurofibrillary tangles and amyloid plaques could directly affect specific regions of the human brain. Earlier, in 1997, Dr. Gerber and colleagues at the Max Plank Institute of Neurobiology's psychiatric department examined histopathological brain sections with F. Johan, a specialist with decades of expertise in human neuro-cellular structure (11). This work has considered as the second documented case of AD. The examination of the tissue slices revealed numerous plaques composed of abnormal protein deposits. This work demonstrated the genomic analysis which can be performed on preserved human brain tissue, offering valuable insights into the molecular basis of AD (12).

A century after Dr. Alzheimer's original invention, his observations continue to be validated clearly demonstrating the structural and pathological differences between healthy brain and those affected by AD. In India, AD is recognized as the sixth leading cause of death (13) and recent statistics suggest that it may soon become the third most common cause of mortality among older adults. This growing prevalence highlights the urgent need for early detection to prevent or slow the progression of the disease. Diagnosing AD involves many clinical assessments and the analysis of the extensive multivariant data, ranging from the clinical examinations and manual evaluations to the imaging interpretations. But it remains complex, time-consuming and highly repetitive. The MRI-based classification plays a crucial role in accurately identifying brain abnormalities, though it presents significant challenges (14). The advances in the interpretations of MRI scans have enabled the development of new techniques capable of supporting earlier and more reliable detection of AD.

The CNN-based methods can identify patterns in human brain MRI scans which offers a promising method for the early detection of AD. Despite, the ongoing research, effective treatment for AD remains unavailable, as reported by the AD association in 2019. In India alone, more than two million individuals are affected and the projections indicates that by 2050 around 10 million people may be living with AD. The disease estimated to impact one person every 67 s (15, 16).

### 1.1 Objectives of the paper

The objectives of the paper are:

1. Analyse a comprehensive literature survey, including the background of the problem, methods adapted for Alzheimer's Disease (AD), and its various stages and types, as evidenced by MRI scans.
2. Develop a Deep Convolutional Neural Network with the Attention mechanism to enhance the model's focus on critical brain regions affected by the disease.
3. Formulation of experimentation on the proposed approach with diversified input parameters, and calculation of the loss and accuracy of the classifications.
4. Finally, by comparing the proposed approach with existing methods like CNN and SVM, we aim to demonstrate that the proposed model achieves high efficiency and low loss

### 1.2 Practical and theoretical implications

The proposed model can act as triage tool in the clinicians' workflows by flagging scans with high probability of AD and progressive MCI. The attention overlays can help the radiologist and neurologist to localize structural biomarkers which can contribute to the prediction, increased clinical trust and correct follow-up. By reducing the unnecessary specialist referrals and prioritizing high-risk patients like automated tool can lower diagnostic delays and resource burden in memory clinics.

From a methodological perspective, the results show that explicit spatial attention improves MCI subclass discrimination. This supports the hypothesis and attention module can increase the model sensitivity to understated and provide a reproducible pipeline for integrating interpretability modules into volumetric neuroimaging classifiers.

### 1.3 Organization of the paper

The rest of the analysis of the paper is organised in the following manner. Section 2 provides a brief overview of Alzheimer's disease, including the identification of the disorder and methods of human brain scanning models and related surveys. Section 3 outlines the methods and materials which are required for the proposed methodology. Section 4 deals with the system model and its algorithm of the proposed work. Section 5 illustrated the experimental setup, results and performance analysis. The conclusion and future work are explained in Section 6.

## 2 Related works

The related works of AD have demonstrated that the disease can be rectified in the beginning stage. Some specific studies have been conducted, with results that clearly demonstrate the difference between AD and healthy controls. Yang et al. (17) have presented a review of many techniques that provide solutions for AD. They have mainly considered four models, of which the first model uses pre-training based on a deep learning architecture. This model uses large datasets and has achieved 89.58% accuracy. The second model uses a full deep learning architecture that extracts features from the linguistically trained datasets. This gives 85.4% accuracy. The third model utilises the VGG-Net architecture to extract the acoustic patterns, achieving a detection value of 78.9%. The final model uses both linguistic and acoustic patterns and gives an accuracy value of 72.92%.

Afzal et al. (18) have identified that the brain pathological changes are the main reason for AD, and the same can be estimated using neuroimaging systems. They have suggested a mechanism to improve the diagnosis of AD using various image processing and machine learning algorithms. The performance metrics were analysed using MRI, fMRI, and PET scanned images. The authors have applied the transfer learning technique on the MRI scanned datasets and achieved 98.41% accuracy. Likewise, by using the SVM classifier, the authors have achieved 92.4% accuracy.

To provide an efficient solution for the AD, Orouskhani et al. (19) found a model using a conditional deep triplet network. The authors have used a deep triplet model, which contains a convolutional layer, a max pooling layer and a fully connected layer. They have utilised MRI data and applied image recognition techniques. They have classified the AD into four categories like no dementia, very mild dementia, mild dementia and moderate AD. The authors have achieved 99.41% of accuracy using the OASIS dataset.

Ebrahimi et al. (20) have developed a model using convolutional neural networks for AD. They have used both 2D and 3D MRI images for their work and developed deep models. The authors used based deep learning model with transfer learning and achieved 96.88% accuracy. However, this method cannot handle larger datasets. The author also detected a solution using sequential deep modelling (21). In this approach, the authors used multiple deep sequences and slice-based voxel models. Additionally, here utilized a temporal convolutional model, achieving 91.78% accuracy. Due to the limited dataset in their proposed work, they have implemented transfer learning.

Liu et al. (22) proposed a plan using a generalizable deep learning model to understand and detect AD. The authors have taken the structural MRIs and used 3D deep convolutional networks. As the model has been developed for large datasets, the authors have developed a two-stage quality checking. The authors have used a gradient boosting model and estimated the accuracy value as 87.59%. This model is the fastest classification model based on the regions of interest.

Liu et al. (23) found a solution for AD using depth-wise separable convolutional neural networks. The authors initially tried the conventional CNN method, achieving an accuracy value of 78.02%. Also, they have classified the images into normal, AD and MCI. Then, along with the CNN, the depth-wise separable convolutional neural networks were combined and implemented. This proposed model gave 87.94% accuracy. For both implementations, the OASIS dataset has been chosen.

Lin et al. (24) have proposed a machine learning model to detect AD. The authors have chosen an ANN model with three input layers and one output layer. They have used the OASIS dataset, which contains 60–96 years of data. According to them, early detection and diagnosis of AD is crucial due to the ageing population. However, timely intervention in this AD may help the patient recover more easily. Using ANN, the authors have achieved 89.7% accuracy.

Mujahid et al. (25) have identified an approach for AD detection which uses an adaptive synthetic technique and a deep learning model. The authors have collected MRI images and utilised adaptive synthesis, a technique primarily employed for object detection, facial expressions, and image analysis. They have used ensemble deep learning with a transfer learning approach to avoid the overfitting problem. This model has achieved 97.35% accuracy, and the authors have compared this with the existing methods.

Odusami et al. (26) have presented a novel approach using a finetuned ResNet18 network. According to the authors, mild cognitive impairment is the first symptom of AD, and they have proposed a deep learning model to identify the same. The ResNet contains 18 layers, which are useful for filters and other layers. They have taken a dataset from fMRI databases and estimated various performance metrics like accuracy, F1-score, precision and recall. The proposed model achieved 95.99% accuracy in the AD classification scenario.

Kong et al. (27) found an approach for AD detection which is based on 3D convolution. The authors have proposed an image fusion technique to combine MRI and PET images, which will help classify normal, AD, and MCI images. They have used a three-layer sparse autoencoder to perform this operation speedily. They have used 3D images and estimated the accuracy value as 89.80%. To further improve the solution, Battineni et al. (28) have employed a multi-model machine learning algorithm. The authors have taken the neurology data from the University of Washington. After the data preprocessing, the authors have tried with random forest algorithm, support vector machine, naïve bayes classifier, logistic regression and gradient boosting algorithms. The authors have concluded that these traditional machine learning algorithms perform well on the given data set to identify the AD and gave an accuracy of 97.58%.

Khare et al. (29) have presented a method for identifying AD using an automated adaptive and explainable learning method. The authors have tried this method with EEG signals and used wavelet transform methods. They have used 10-fold validations and obtained 97.85% accuracy. However, the issue with this proposed model is that the authors have used only 23 subjective datapoints to support it. So, it may not be support properly while testing with large datasets.

Chui et al. (30) have well known the importance of convolutional neural networks and transfer learning. So, they have used these techniques in the detection of AD using MRI scans. In addition, they have used a generative adversarial network to train the minority classes of data. The layers of CNN are used to find and classify the AD promptly. By this, the authors have achieved 91.6% accuracy. Petersen et al. (31) presented a solution for AD using biomarkers. Also, they have used Down syndrome, which is a common genetic cause of the inability. They have considered the longitudinal changes in the biomarkers. The variations in the detection of AD have been reviewed again. They have attained an accuracy value of 86.8%.

Cheung et al. (32) have proposed an AD detection model using deep learning algorithms which uses retinal images. The batch normalisation method has been applied to the images during the feature selection process. They have estimated 89.6% accuracy. Venugopalan et al. (33) have also proposed a multimodal deep learning algorithm for early detection of AD. The authors have used shallow learning models, which are a part of deep learning models. They have used the ADNI database for the dataset, which contains MRI images, PET and biological markers. Also, they have used 3D CNN with the dimensions of 22 × 23 × 18, which gives better accuracy of 86%.

Gao et al. (34) also addressed the AD detection. They have implemented the solution using a deep learning model, and they have achieved good performance. They have frequently used a patch-based algorithm and regions of interest. They have compared the advantages and disadvantages of voxel-based, slice-based, ROI-based and patch-based feature extraction methods. After feature extraction, they utilised several supervised and unsupervised learning algorithms, including CNN. They have obtained the accuracy value of 91.40%.

For the early detection of AD, Safi and Safi (35) used a model based on EEG signals. The authors have used Hjorth attributes, which are essentially nonlinear features derived from statistical analysis. These attributes will be derived from the EEG signals, which use wavelet transforms. They have used the KNN and SVM algorithms for the AD classification. They have achieved 97.64% accuracy with the KNN algorithm and 95.79% using the SVM algorithm.

Marwa et al. (36) have analysed the AD problem using MRI images and employed a deep learning model for detection. In their proposed deep learning architecture, they have used the conventional 2D CNN, which contains five layers. They have taken the batch size of the input data as 40 and used 100 epochs. They have estimated 100% sensitivity and 95.68% accuracy.

Arafa et al. (37) discussed and presented a model for AD detection using deep learning approaches. They have clearly depicted the importance of machine learning and deep learning techniques in identifying AD. After the data preprocessing stage, the authors applied several image processing techniques, including intensity normalization and contrast enhancement. They have used convolutional neural networks, deep neural networks and recurrent neural network models for the classification process. Mahendran et al. (38) have proposed a model using a deep learning framework. The authors have used a deep recurrent neural network and an enhanced deep recurrent neural network. They have used an embedded-based feature selection like LASSO regression. They have taken the GEO Omnibus dataset and estimated the accuracy value to be 80.4%.

Kishore, P et al. (39) have reviewed the machine learning models for AD detection. The authors have highlighted the differences between a healthy brain and an AD brain. They have utilized MRI-scanned images and employed the SVM algorithm for classification. They have conducted an exploratory data analysis and calculated the minimum value, maximum value and mean of the featured data. Sharma et al. (40) have presented an AD detection method using a VGG16 extractor. The authors have proposed a CNN model using MRI scans and used VGG16 for feature extraction, which is a CNN-based pre-trained model. They have calculated that the accuracy of this proposed study is 90.4%.

El-Sappagh, Shaker et al. (41) have tried to evolve the automatic detection of AD. They have used ensemble learning with multimodal time series data for AD detection. The feature selection process will be conducted using statistical analysis. They have used the ADNI dataset for this study and calculated the information gain value. The raw time series data analysis, data preprocessing, statistical feature extraction, feature selection and classification are major steps of the proposed algorithm (42). Base model generation, ensemble pruning and ensemble integration are the major steps of the ensemble learning. They have obtained 96.5% accuracy with the proposed model.

Komal et al. (43), have classified the AD using advanced CNN algorithm and MRI images. The authors have used densely connected neural networks to extract the pre-processed images. The proposed architecture contains five levels of neural networks and attention maps. This will process the 224 × 224 pixels of MRI images through convolutional and pooling layers. In addition, the batch normalization and ReLU activation function are used in the proposed model to minimize the computational load and speed convergence. The authors have used ADNI dataset and they have achieved 98.40% of accuracy. Arjun Kidavunil Paduvilan et al. (44), were designed a hybrid deep learning model using SVM algorithm for the early AD diagnosis. In their proposed work, they have used two traditional deep learning model namely GoogLeNet and DenseNet-121. They have also used feed forward neural network to identify the AD class as very mild person living with dementia, non-person living with dementia, moderate-person living with dementia and mil-person living with dementia. The authors have used ADNI datasets which contains MRI scans and PET scans. This model has achieved 98.5% accuracy and 98% of F1-score.

Upon summarizing the prevailing relevant literature, it has been discovered the following technical deficiencies and the gaps in the existing research.

1. Most existing models have chosen a few benchmarked datasets, such as ADNI, which provide real-world, world-diversified data that includes diverse age groups and ethnicities. So, the dataset like OASIS can be chosen to solve this issue.
2. Since many existing models are based on static MRI-scanned images, they cannot effectively identify and adapt to changes in brain images. This leads to critical tracking of disease progression.
3. Many models continue to provide outputs as black boxes, lacking sufficient insight for clinical trust.
4. The existing approaches struggle to classify MCI and distinguish it from aging factor, presenting a significant challenge due to difficulties in creating current models.
5. The accuracy of diagnosis in the training datasets is dependent on clinical labels, which can have subjectivity and variability affecting model training quality.

The proposed model should consider a diversified dataset to accommodate a variety of input data. Instead of a conventional CNN, a Deep CNN can be chosen to increase the number of layers. If the number of layers is increased, then the accuracy of the diversified input data can be considerably improved. The attention mechanism can be added along with the deep CNN to understand and implement the relationship between the extracted features from the input data.

## 3 Materials and methods

The detection of AD involved the combination of several factors, including clinical assessments, biomarker analysis data, neuroimaging and mainly machine learning algorithms. The materials and methods for this study have been chosen very carefully to avoid errors and to achieve high efficiency.

### 3.1 Materials

#### 3.1.1 Dataset description

The OASIS, which stands for Open Access Series of Imaging Series which is a database that consists of MRI data of the brain. The primary intention of this database is to create a repository for the scientific community. It has been developed to facilitate progress in the basic and clinical neurosciences. Dr. Randy Buckner, a senior scientist at The Howard Hughes Medical Institute, Haward University, initiated this initiative. This dataset consists of two different types of images, namely Cross-sectional MRI data and Longitudinal MRI data.

The study utilizes 834 unique records with a total of 1,042 MRI scans. All these subjects are handed properly with 5 multiclass to differentiate the stages progress towards cognitive decline and it include all the genders. Especially, around 834 subjects are more than 60 in age which are clinically diagnosed with very mild to moderate AD. The dataset consists of unique subjects and unique MRI sessions. All subjects were handled at the subject level and each subject's scans all the derived 2D slices were kept together in a single split to avoid any subject level data leakage. In each class, subjects have been scanned a minimum of two times, and they have been separated by at least one year. Likewise, the database has 1,042 images, and they are right-handed for both genders. Among these 210 subjects, who were found to be non-person living with dementia by the study, and another 350 subjects, who were found to be person living with dementia. Further, 145 subjects were identified and grouped as having mild to moderate AD and Early, Late MCI as 68,61. For the 2D experiments, the axial slices were extracted the hippocampal and medical temporal lobe region which can be used for volumetric experiments. They can be resampled volumes at 1 mm × 1 mm × 1 mm isotropic resolution. The Table 1 represents the dataset description of class and samples.

**Table 1:** Dataset description with sample details.

Class	Subjects used	Samples after preprocessing
Alzheimer disease (AD)	210	210
Cognitively normal (CN)	350	350
Mild cognitive impairment (MCI)	145	145
Early MCI	68	68
Late MCI	61	61

### 3.2 Methods

The dataset chosen for this work is widely used in AD research. It consists of a combination of MRI images and patient clinical data, which will help apply machine learning, statistical analysis, and preprocessing. For our study, we have chosen the OASIS-3 version, which can be downloaded from https://sites.wustl.edu/oasisbrains/. These are the T1-weighted MRI scan data with clinical data in the form of a.csv file. This file will impute missing numerical values using the mean of the specific attribute. The categorical values will be encoded using a hot-encoder or the labelled encoder. For the normalisation of the value, the Min-Max scalar will be used.

The structural T1-weighted MRI scans were processed using the standard neuroimaging tools only. As it is the combination of scanned images and clinical data, the preprocessing pipeline includes several steps (45).

• Format conversion and loading—The MRI scans in the T1 format will be loaded using Python libraries.
• Reorientation and skull stripping—The MRI images will be reoriented to regular anatomical planes. Also, the non-brain tissue images will be removed using the PET tool.
• Spatial Normalisation—To achieve consistent spatial orientation and alignment across all the subjects, all the images will be registered to the MNI152 template space, which the International Consortium of Brain Mapping developed.
• Resampling and Intensity Normalisation—The images will be resampled to an isotropic voxel size of 1 × 1 × 1 mm^3^. These intensity values will usually be normalised using z-score standardisation. This will be used for inter-scan variability.
• Dimensionality reduction—This is an optional step that can be useful for computational efficiency. The relevant images will be sliced into a 1D array using principal component analysis.

The MRI preprocessing has followed this deterministic pipeline steps to complete the Preprocessing. The skull-stripping with FSL- BET and the rigid + affine registration to MNI152 can resample the MRI sessions into 1 mm × 1 mm × 1 mm voxels. The per-volume z-score intensity normalization also used for the data preprocessing. All the preprocessing steps are reproducible. After data preprocessing, the dataset will be split into a training dataset and a testing dataset in a 70:30 ratio. This ratio is chosen to preserve the distribution of diagnostic levels and prevent overfitting and underfitting issues in the dataset.

### 3.3 Proposed methodology

#### 3.3.1 Deep convolutional neural networks (DCNN)

The first and most important part of the DCNN design is the input layer. Its primary function is to take in the raw data—in this example, an image—and get it ready for the neural network to process it further (46).

##### 3.3.1.1 Input layer

An essential part of a neural network, the input layer is in charge of taking in raw data and getting it ready for additional processing. The input layer in this instance is configured to accept pictures of dimensions *H* × *W* × *C* that are represented by a tensor *I* in Equation 1:

$$I\in R^{150\times 150\times 3}$$ (1)

##### 3.3.1.2 Convolutional layer

The core building blocks of DCNN, which are used to extract spatial characteristics from input data like pictures, are convolutional layers. To create feature maps—which draw attention to intricate structures, edges, and textures in the input—a sliding filter, or kernel, is applied across the input data. Convolution is used for feature extraction expressed in Equation 2:

$$F(x,y)=\sigma \left(\sum_{i=0}^{K-1}\sum_{\;j=0}^{K-1}I(x+i,j+j)\cdot W(i,j)+b\right)$$ (2)

where *x* and *y* are the top-left coordinates of the kernel's current location, and *I* (*x* + *i*, *y* + *j*): Indicates the value of the input at position (*x* + *i*, *y* + *j*). The weight of the kernel (filter) at location (*i*, *j*) is indicated by the symbol *W* (*i*, *j*). The dimensions of the kernel are *K* × *K*. The bias term, which aids in the model's learning of offsets in the data, is introduced to the convolution procedure. *σ*: The activation function used to add non-linearity to the output, such as ReLU (Rectified Linear Unit).

##### 3.3.1.3 Pooling layers

The DCNN frequently employ max pooling, a down-sampling technique, to shrink feature maps' spatial dimensions while keeping the most noticeable features. Max pooling makes the model more resilient and efficient by choosing the largest value from a specified window or section of the input matrix, preserving the most important feature in that area. The max pooling operation with a pooling window of size *P* × *P* and an input feature map *F* (*x*, *y*) may be written as follows in Equation 3:

P(x,y)=max{F(i,y)0≤i,j<P} (3)

A window with dimensions *P* × *P* (e.g., 2 × 2 or 3 × 3) is specified. This window, which is usually non-overlapping, moves over the input feature map with a given stride (step size). The pooling procedure chooses the highest value from the window's covered *P* × *P* region at each stage. A new feature map with smaller spatial dimensions is created from the pooled data that are produced. In the event when the stride is equal to the pooling size *P*, for example, the output's height and breadth are lowered by a factor of *P*. In DCNNs, max pooling is a crucial function that improves efficiency while maintaining the essential information for further processing.

##### 3.3.1.4 Flatten and fully connected layers

Multidimensional arrays make up the feature maps (F_att) produced by earlier layers, such as convolutional or attention layers. These feature maps, which are frequently expressed as tensors with dimensions [*H*, *W*, *C*], extract spatial and depth-wise information from the input data. Where H is the map height, W is the width of the map, and C is the filter.

These feature maps need to be flattened into a one-dimensional vector to be processed in dense (completely linked) layers. The representation of this operation in Equation 4:

$$\vartheta =\mathrm{Flatten}(F_{\mathrm{att}})$$ (4)

Here, the tensor is rearranged using the Flatten Flatten operation to create a vector (*ϑ*) whose length is equal to the entire number of elements in *F*_att, or *H* × *W* × *C*. Followed by dense layers:

The vector (*ϑ*) is sent into a number of dense layers for additional processing after it has been flattened. Dense layers convert the input vector and extract high-level patterns using learnable weights and biases. The dense layer can be expressed mathematically as follows in Equation 5:

h=σ(W⋅ϑ+b) (5)

where *W* as weight matrix, *σ* as activation function, *ϑ* as vector, *b* as bias, *h* as output.

Closes the gap between decision-making processes (in dense layers) and the extraction of spatial features (from convolutional layers). Discover intricate connections between the ultimate task—such as classification or prediction—and the characteristics that were extracted. Enhances the network's capacity to generalise to unknown inputs by ensuring that it can represent non-linear patterns in the data.

##### 3.3.1.5 Output layer

**Figure 1:**
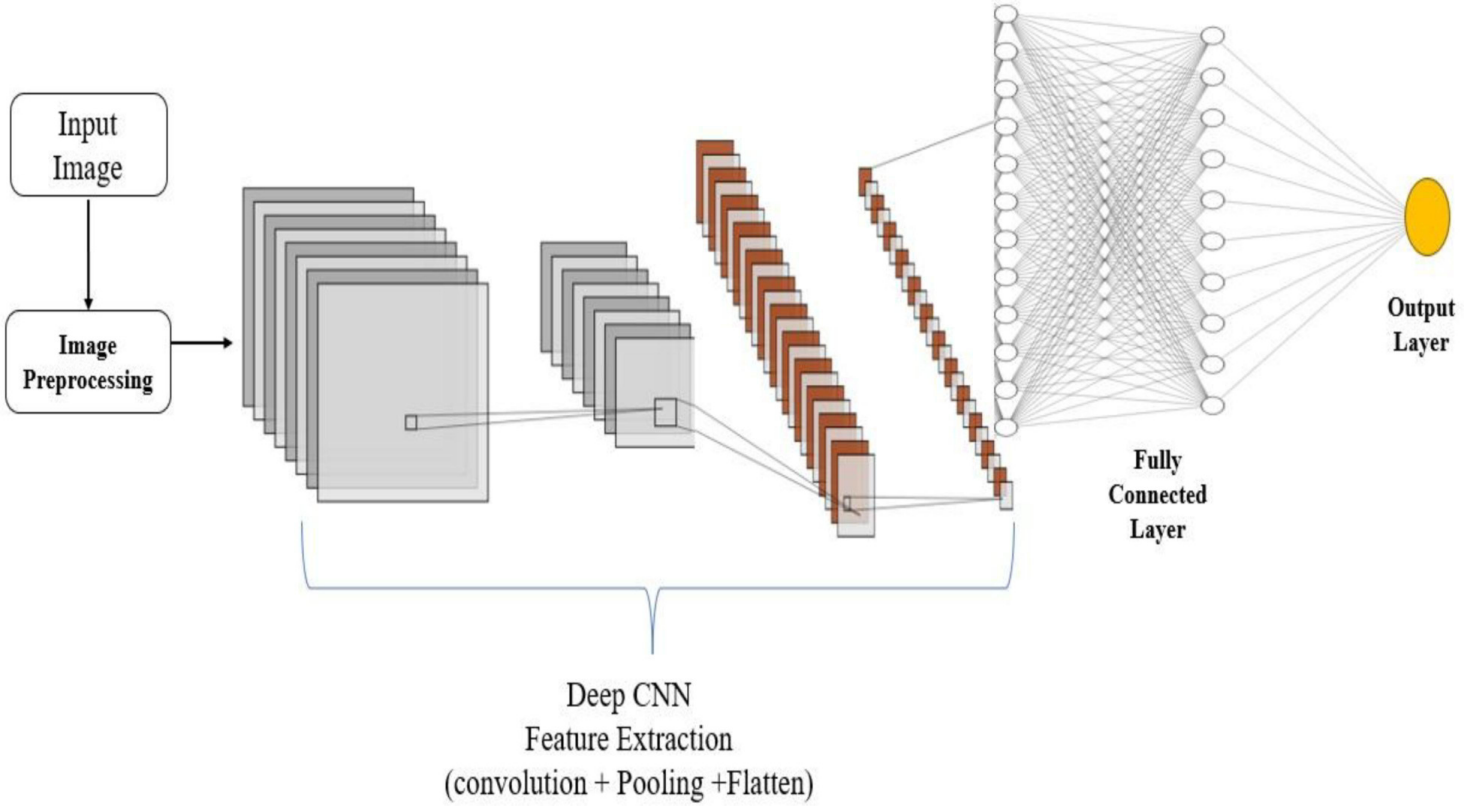
Flow diagram of the deep convolutional neural networks.

The last dense (completely connected) layer of a neural network is in charge of producing output probabilities for every class in a classification job (47). The SoftMax activation function, which normalizes the dense layer's outputs into a probability distribution across the classes, is used to do this. The final dense layer produces class probabilities using a SoftMax activation in Equation 6:

$$P(y=c|x)=\frac{\exp\;(h_{c})}{\sum_{k}\exp\;(h_{k})}$$ (6)

where *hc* as (logit) assigned by the dense layer, *hk* as scores for all possible classes *k*, *P*(*y* = *c*|*x*) as probability of the input *x* being classified as class *c*, exp as exponential function. The Figure 1 illustrates the schematic flow of the proposed work.

#### 3.3.2 Attention mechanism

A crucial element that concentrates the model's processing capacity on the most significant spatial areas of a feature map is the attention block. Each region's priority is constantly assigned according to how relevant it is to the job at hand, such as segmentation or classification. The spatial attention module can be place after the final convolutional block of the Deep-CNN. Let $F\epsilon R^{H\times W\times C}$ is the feature map output by the last convolutional block. The attention module computes a map $A\epsilon [0,1{]}^{H\times W}$ and it will return an enhanced feature *F*_att_ by using the elementwise multiplication operation expressed in Equations 7–9 (48).

$$S=\mathrm{Con}v_{1\times 1}(F;W_{s})\epsilon R^{H\times W\times 1}$$ (7)

$$A=\sigma (\mathrm{Batchnorm}(S)+b)\epsilon [0,1{]}^{H\times W}$$ (8)

$$F_{\mathrm{att}}(x,y,c)=F(x,y,c)\cdot A(x,y)$$ (9)

where $\mathrm{Con}v_{1\times 1}$ indicates 1 × 1 convolution which computes scalar relevance score at each spatial location, *σ* indicates the sigmoid activation and Batchnorm indicates stabilized training. The 1 × 1 convolution has output channels=1 and it will be followed by a sigmoid to yield spatial attention weights. Finally, $F_{\mathrm{att}}$ is fed to global average pooling and dense classification head. The combination of channel and spatial attention variant are also evaluated by convolutional block attention module and found the spatial-only module provides a good trade-off between performance and interpretability.

The Spatial attention emphases the classifier on brain which may vary with diseases while the channel attention biases rather than locations. For the MRI inputs and the clinical goals, the spatial attention module produced clearer attention overlays for clinical interpretation.

### 3.4 System model

The Deep CNN have confirmed unconditional support in the medical image processing task, including AD detection. This can be performed using the structural MRI scanned images and optimal machine learning algorithms. Additionally, traditional CNNs may not be optimal for brain-related images affected by AD. To address this limitation, this study utilised a deep CNN, also known as an ensemble-based CNN. In this work, the number of layers in the neural networks can be improved so that the feeding of each layer can be increased, and accurate learning can be possible. In addition, an attention mechanism has been incorporated in the proposed work to direct the model and to prioritise the features to the layers by which the accuracy can be increased.

**Figure 2:**
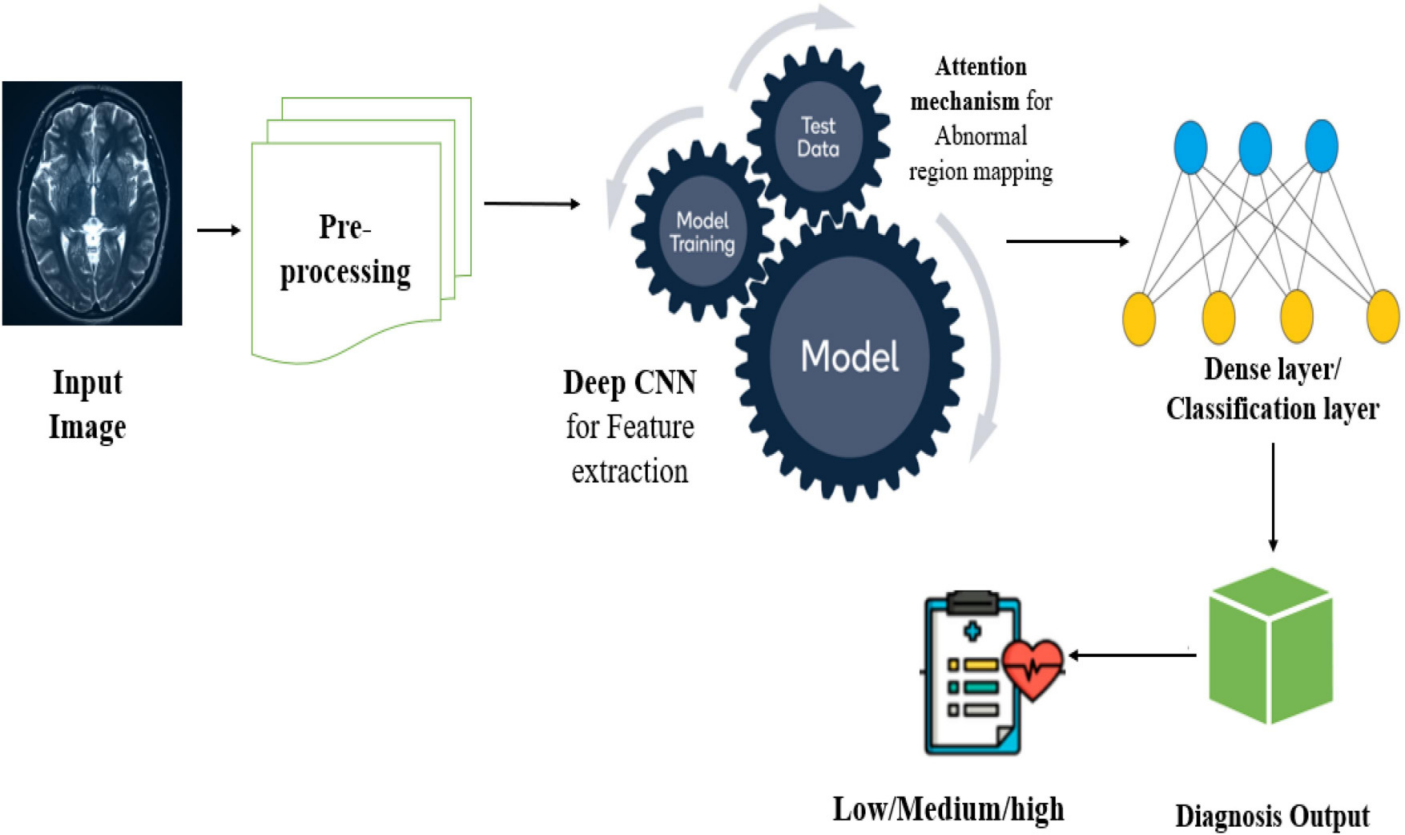
Architecture of the proposed work.

The Figure 2 illustrates the architecture of the proposed work, which combines both Deep CNN and the Attention mechanism. The Deep CNN, similar to VGG-16, is utilised to extract high-level spatial features from MRI images. Typically, CNN layers learn hierarchical feature representations automatically. In contrast, Deep CNNs learn features through multiple layers, including brain tissue boundaries, abnormalities in brain structure, and other patterns. These features are highly associated with the AD detection (49). The attention mechanism, which can easily find the relevance of the input features, will be applied on top of the feature maps generated by the CNN. This allows the network to focus uniquely on important sections of the brain, like the entorhinal cortex, ventricles and hippocampus, because these parts are identified as implicated in the AD development.

The most important benefit of using the attention mechanism along with Deep CNN is to enhance the interpretability of the system by enlightening the brain regions for decision-making. This will be helpful for the classification with low noise. Also, it allows the system to handle the changes in the early stages of the AD.

The proposed system, Deep CNN with attention mechanism, is organized into four components, namely data processing, feature extraction, attention-based feature extraction and classification. Each phase is very complicated to achieve high accuracy. The input of the model is pre-processed brain MRI images, which are derived from the OASIS dataset. This preprocessing includes skull stripping, intensity normalization, spatial normalization and resizing of the images. The suitable shape for the input Deep CNN is 224 × 224 × 224 or 128 × 128 × 128 voxels (50).

The proposed model is a deep CNN enhanced with an attention mechanism, designed for both 2D slice-based and 3D volume-based experiments. The 2D architecture takes a 150 × 150 × 3 axial slice as input and process it through three convolutional blocks with 32,64 and 124 filters respectively. Each block contains two 3 × 3 convolutions with a batch normalization and Leaky ReLU activation followed by 2 × 2 max pooling. An attention module is then applied where a 1 × 1 convolution generates attention logits which are passed through sigmoid function to produce an attention map that is multiplied elementwise with the feature map. The final features are fed into a classification head comprising global average pooling, a 512-unit dense layer with Leaky ReLU and 0.5 dropout and a final 5-unit softmax layer. Similarly for 3D experiments, the same architecture is adapted by replacing the 2D operations with their 3D counterparts and using a 128 × 128 × 128 × 1 input volume. All the convolutional layers employ L2 regularization with a weight decay of 1 × 10^−4^.

The Deep CNN is used as a feature extractor. The ReLU activation function will be introduced along with the VGG-16 to introduce the non-linearity into the proposed model. Assume the proposed model consists of *n* shallow ReLU networks with 2n neurons; then the network function will be defined as in Equation 10

f(x):=ReLU(2x)−ReLU(4x−2)+ReLU(2x−2) (10)

where *x* is the first kernel coordinator and represents the feature, this process concatenates the remaining features multiple times based on the additional layers in the DCNN. So, the model can be extended to the ReLu function with the linear unit in the same situation in Equation 11.

$$\mathrm{ReL}U^{N}=\mathrm{ReLU}\;o\;\mathrm{ReLU}\;o\ldots o\;\mathrm{ReLU}$$ (11)

Assume ReLu (*x*): = max (0, *x*). This function is not depending on the number of layers in the Deep CNN. Conceptually, the (Equation 12) can be interpreted using its derivatives in Equation 12.

$$\frac{\partial }{\partial a}\mathrm{ReL}U^{N}(x)=\{\begin{array}{cc} 1 & \mathrm{if}\;x>0\\ 0 & \mathrm{else} \end{array}$$ (12)

The proposed model offers a significant solution to the dying ReLU function, which occurs when a large number of neurons become idle and their output is always zero. This will create poor initialization, high learning rates and large negative bias shifts. This can be avoided using the following Equations 13, 14.

$$\frac{d\mathrm{ReLU}(x)}{dx}=1\;\mathrm{for}\;\mathrm{positive}\;\mathrm{inputs}$$ (13)

$$\frac{d\mathrm{ReLU}(x)}{dx}=0\;\mathrm{for}\;\mathrm{negative}\;\mathrm{inputs}$$ (14)

The proposed method uses the leaky ReLU to avoid this issue. According to this, instead of outputting zero for negative inputs, it will give a small slope in the output. This can be expressed as follows in Equation 15.

$$\mathrm{LeakyReLu}\;(x)=\{\begin{array}{cc} x & \;\mathrm{if}\;x>0\\ \alpha x & \;\mathrm{if}\;x\leq 0 \end{array}$$ (15)

where *α* is the small positive constant and the value is 0.01.

### 3.5 Deep CNN + attention mechanism algorithm

Below the proposed model algorithm is defines follows in Algorithm 1:

Algorithm 1 **Deep CNN + Attention mechanism.**

Input: MRI image data as I from OASIS dataset.

Output: Classification of AD as normal/AD

1. Begin

2.  Load MRI data

3.  Apply Skull Stripping on dataset to remove unwanted regions

4.  Preform Normalization on dataset

5.  Resize image as [H, W, C], ← (150,150,3)

6.  Normalize values $I\in R^{150\;\times 150\;\times 3}$

7. Initialize Deep CNN model

8.  **For** each convolutional Block **do**

9. Convolution $F(x,y)=\sigma \left(\sum_{i=0}^{K-1}\sum_{\;j=0}^{K-1}I(x+i,j+j)\cdot W(i,j)+b\right)$;

10.  Relu → Activation Function;

11.  apply max pooling max{F(i,y)|0≤i,j<P};

12. End **For**

13. Feature map for convolutional layer output

14. Apply attention mechanism:

15.  Compute weights

16.  Generate Attention Map;

17. Flatten Attention map $\vartheta =\mathrm{Flatten}(F_{\mathrm{att}})$

18. **For** each dense layer **do**

19.  Compute activation from hidden layer;

20. **End For**

21. Apply final output with SoftMax activation function

22.  return probability of output

23.   $\mathrm{LeakyReLu}\;(x)=\{\begin{array}{cc} x & \mathrm{if}\;x>0\\ \alpha x & \mathrm{if}\;x\leq 0 \end{array}\;$

24.  Final prediction

25. *End Algorithm*

## 4 Experimentation results and analysis

This section explains the experimental setup for the proposed work, results and the performance metrics. As the classification of the brain images is involved in this study, the accuracy of the classification must be calculated. In addition, the accuracy of the proposed model has been compared with a few existing methods.

### 4.1 Experimental setup

The Table 2 represents the mandatory training parameters required for the proper execution of the proposed methodology. The research is processed with standalone system intel i5 process with integrated intel graphics with 16GB RAM and training is processed with google colab IDE with T4 12GB RAM and library like TensorFlow, PyCharm for visualization and effective training and testing.

**Table 2:** Training parameters.

S No	Parameter	Count
1	Batch size	32
2	Input image size	150 × 150 × 3 2D slice
3	Epochs	50
4	Optimizer	Adam
5	Loss function	Categorical
6	Metric	Accuracy, precision, recall, F1 score for each class
7	Regularizer	L2

For testing purposes, the dataset will be split into 70:30 for training and testing. Using the rescaling technique, the pixel value will be normalised between 0 and 1 with online augmentation with rotations of ±10. The hypothetical dataset contains major classes like AD, MCI and CN. So, 1,042 MRI images will be assigned for testing. The complete model will be trained with a batch size of 32, and 150 × 150 single channel attention map to map all features that feature processed towards final classifier 5 SoftMax for each cognitive disease stage (51, 52).

#### 4.1.1 Training and hyperparameter

The models were trained with the Adam optimizer with an initial learning rate of 1 × 10^−4^ and L2 weight decay of 1 × 10^−5^. The categorical entropy loss method has used and the batch size of 32 has used for early stopping on validation loss. The Resuce LT on Plateau scheduler has used to decrease the learning rate by a factor of 0.5 after 3 epochs without improvement. There are few random seeds like NumPy, framework RNG and, etc. to 42 to enable reproducibility. All these augmentations were applied only during training time.

### 4.2 Results

**Figure 3:**
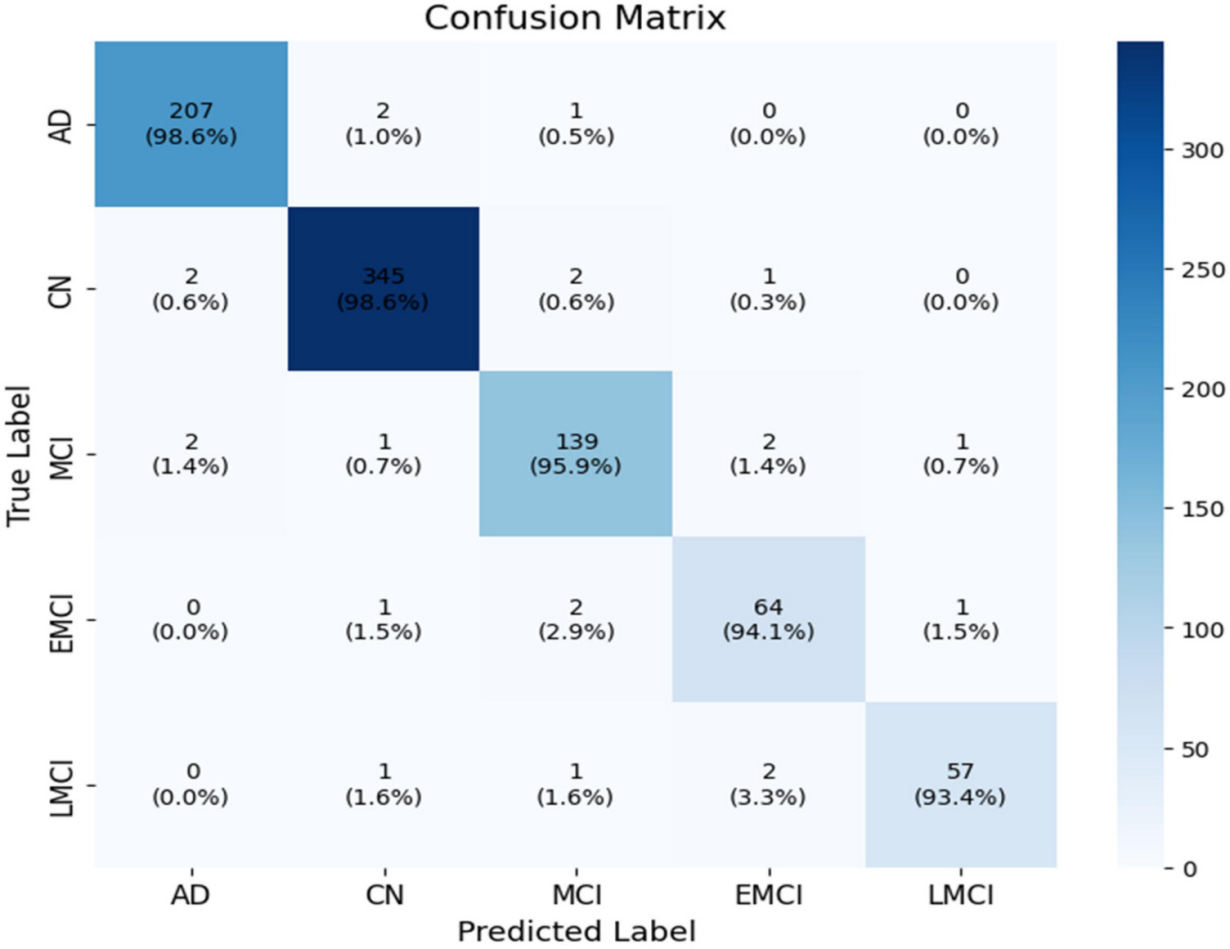
Confusion matrix.

The classification of the proposed model contains 5 classes, namely Alzheimer's Disease (AD), cognitively normal (CN), Mild Cognitive Impairment (MCI), Early Mild Cognitive Impairment (EMCI) and Late Mild Cognitive Impairment (LMCI). When there are multiple classification classes, such as 5, the concept can be effectively illustrated using a multi-class confusion matrix, as depicted in Figure 3.

From Figure 3, it is clearly evidenced that the attention mechanism has supported improving the class discrimination. The AD has predicted with high accuracy. Few AD patients have been predicted as a CN, because of age-related changes which may mimic the CN patterns (52). The MCI has shown slightly better specifically. Overall, the proposed model has proved that the enhancement of CNN and attention mechanisms can focus on relevant features.

**Figure 4:**
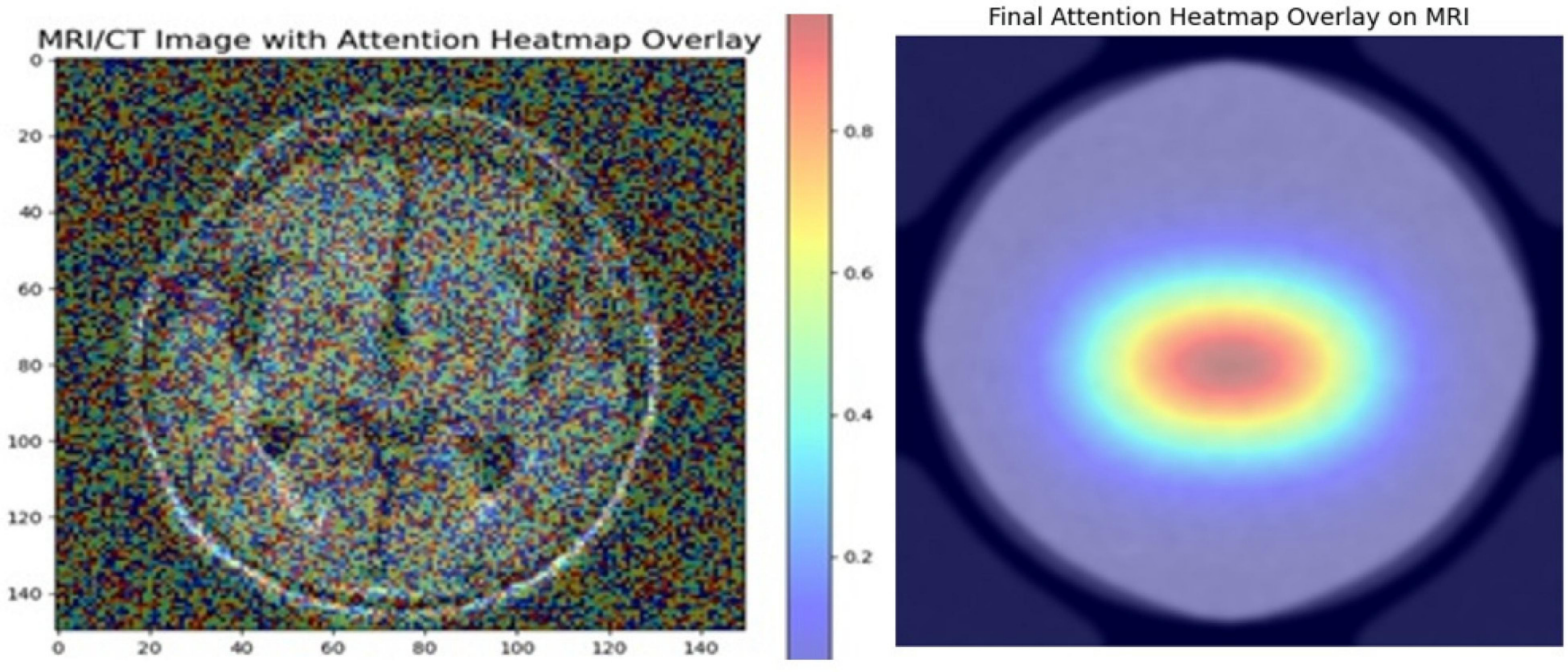
AD predicted heatmap overlay.

Like the Confusion matrix, the heatmap is also useful for data visualisation. In a heatmap, the numerical data will be depicted using different colours. The Figure 4 illustrates the heatmap of the proposed study.

In Figure 4, the rows represent the attention heatmap overly, and the columns represent the predicted class labels. Final attention heatmap show properly identified heatmap overlay on classified samples. This can be used to illustrate how well the model performs in differentiating between different AD classes.

## 5 Performance metrics

**Figure 5:**
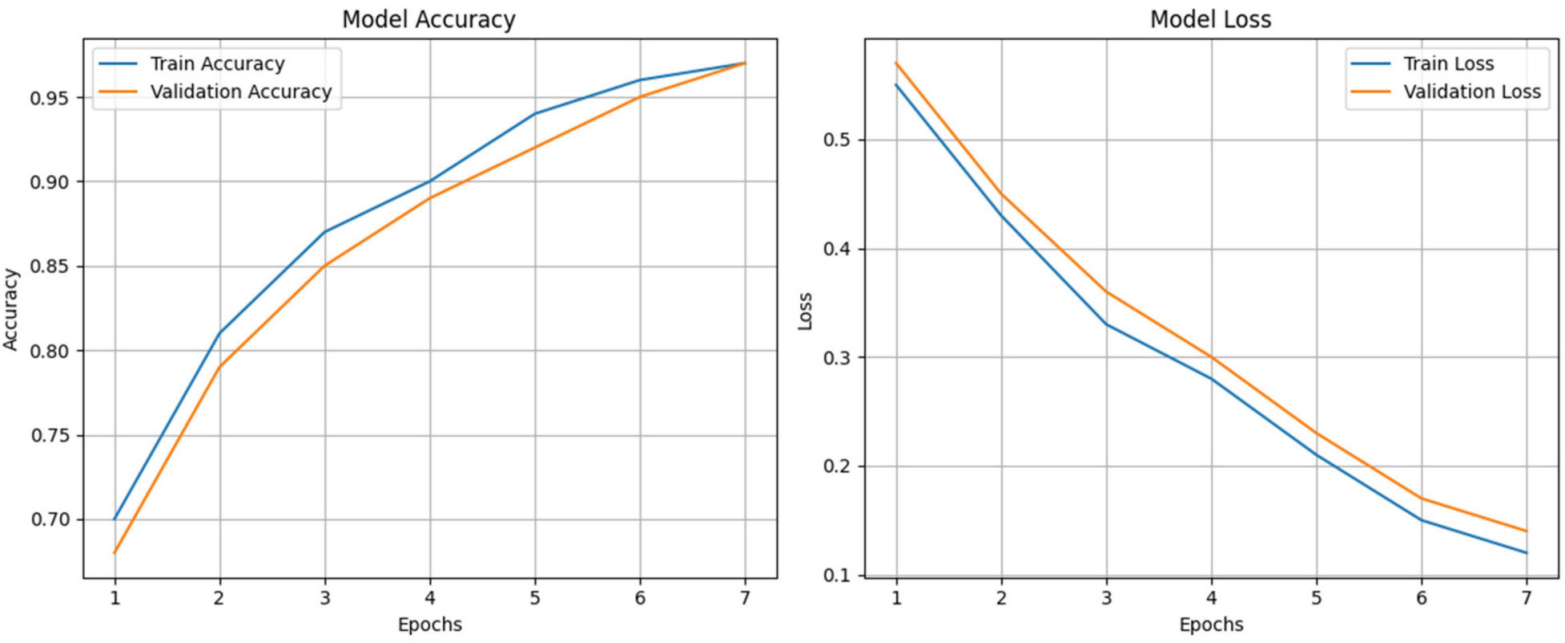
Model accuracy and loss function.

The attention mechanism helps to identify the MCI, specifically, which overlaps both AD and CN in the earlier stages. This will lead to high accuracy and lower loss values. The proposed model calculates both accuracy and loss function, and it is plotted in Figure 5. The formula for accuracy is expressed in (Equation 16).

$$\mathrm{Accuracy}=\frac{TP+TN}{TP+TN+FP+FN}$$ (16)

Figure 5 shows the training and validation accuracy and loss averaged over the five cross-validation folds with shaded regions which indicates ±1 standard deviation. In both training and testing times, the accuracy values are high. For the given dataset, the proposed model has achieved 97% accuracy. Figure 5 depicts the loss function, plotted against the loss value and the epochs. As the loss function is used for categorial cross-entropy, the gap between training and validation is small, and it ensures there is no overfitting of the data.

The Receiver Operating Characteristics (ROC) curve is used to evaluate the classification tasks. It is a very crucial task which can be used for independent threshold evaluation, imbalanced datasets, etc. It can offer a complete and balanced view of the classifier's performance, which may be crucial for risk-sensitive domains like healthcare.

**Figure 6:**
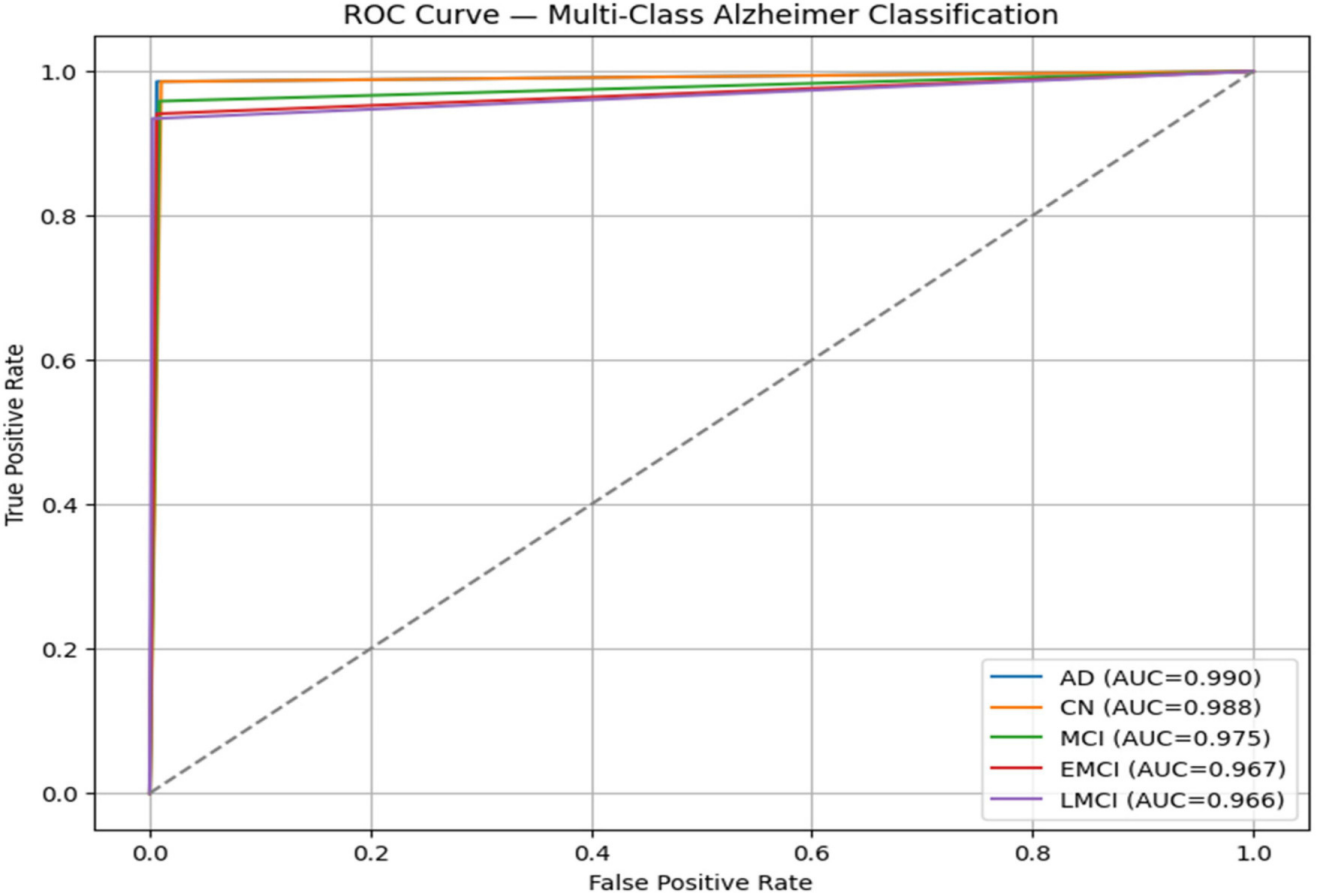
ROC curve.

The Figure 6 illustrates the ROC curve for AD using Deep CNN and the Attention mechanism. Each colour represents a different class, such as AD, CN, and MCI (both EMCI and LMCI). The graph has highly suggested the AD and moderate to the MCI and CN.

When compared to CNN, the Deep CNN has a unique advantage: the number of layers in each block is high, and it captures both low-level and high-level features across layers. Also, this provides higher sensitivity to small disease-relevant regions with reduced training time.

**Figure 7:**
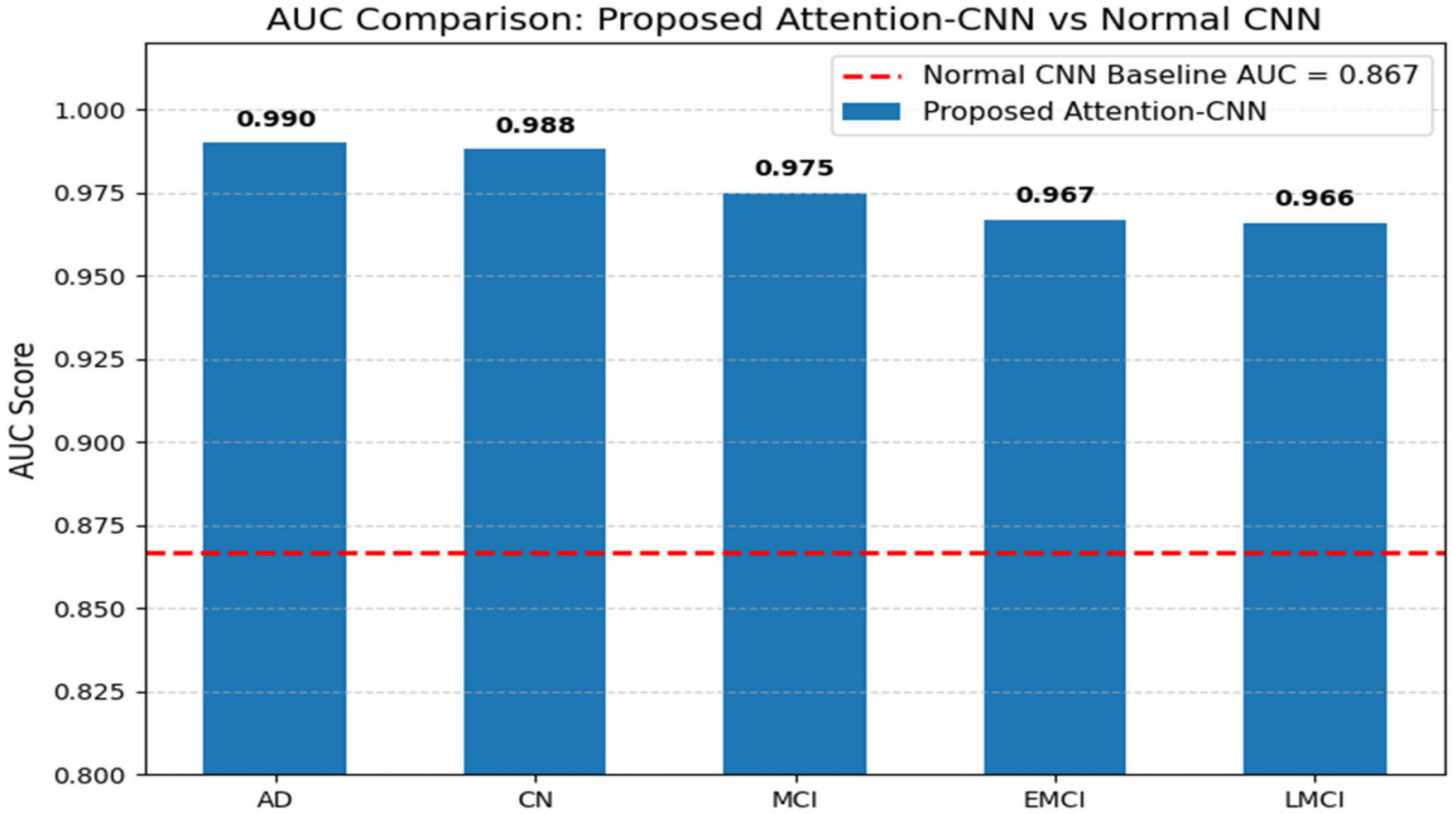
Conventional CNN vs. deep CNN.

Figure 7 illustrates the AUC scores of the Conventional CNN and Deep CNN. The conventional CNN is good for basic classification tasks with small datasets and other resolutions. The proposed Deep CNN + Attention mechanism is more powerful for AD detection, specifically in the early stage, with good accuracy.

**Figure 8:**
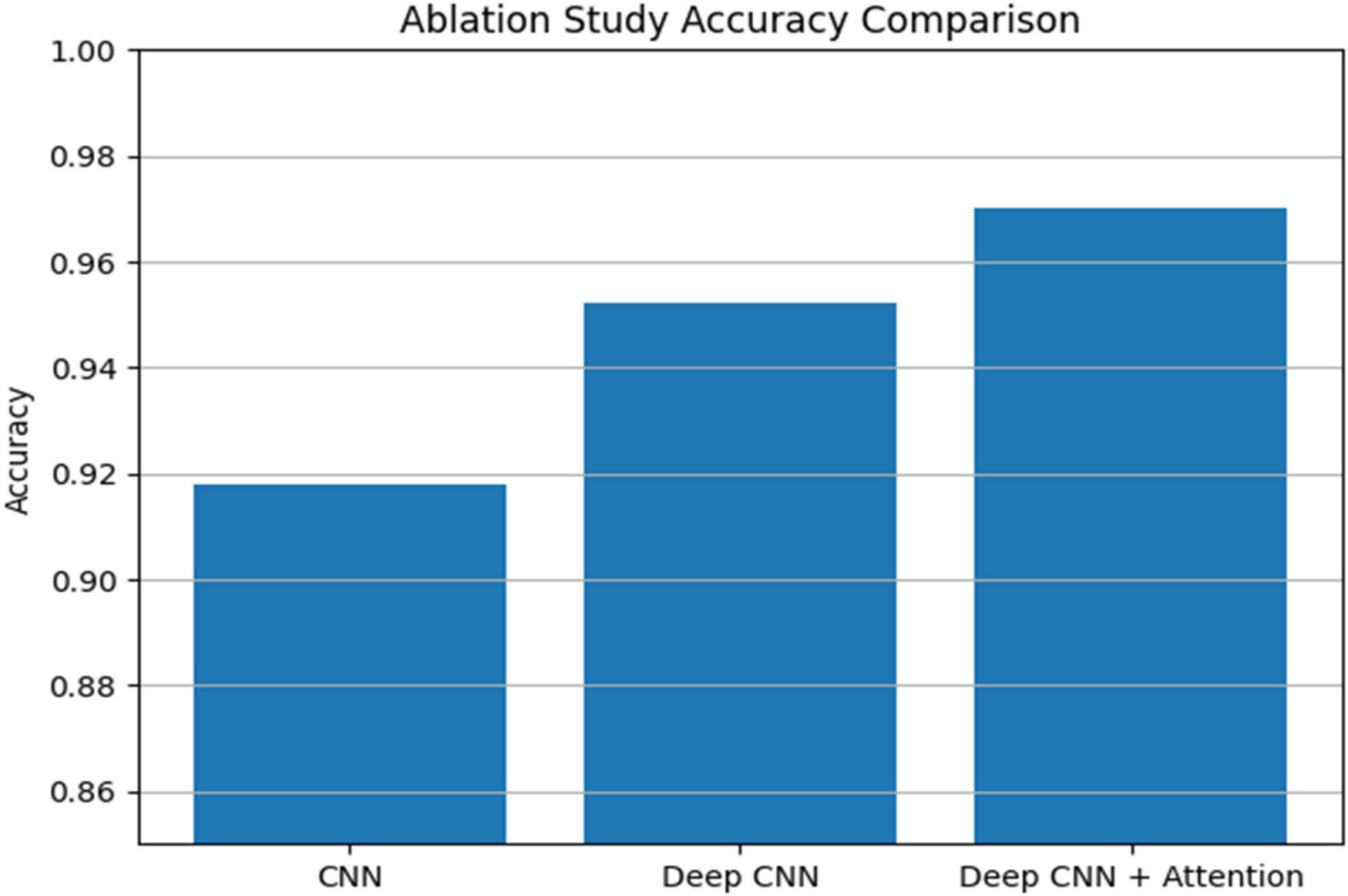
Ablation study.

The Figure 8 illustrates the ablation study which compares the accuracy of three model variants: basic CNN, Deep CNN and deep CNN enhanced with attention mechanism. The results are clearly show that each architectural improvement leads to measurable performance gain. The basic CNN achieved 95.2% which represents additional convolutional layers help the model learn richer and more discriminative features. The best performance has given by deep CNN enhanced with attention mechanism which gives accuracy of 97%. This improvement suggests that the attention mechanism effectively highlights the most informative regions of the input and improve the network ability to focus on important patterns.

### 5.1 Proposed method performance analysis

The proposed study employs a deep CNN enhanced with an attention mechanism which demonstrates high reliability than several traditional prediction approaches used for image-based AD. The brain-extraction process applied in this work utilizes seven specific parameters and incorporates tools suitable for preparations of MRI, DTI and fMRI neuroimaging datasets, along with guidance on how these datasets can be assessed. In differentiating AD from the normal controls, the proposed model achieved an accuracy of 97% for AD classification.

**Figure 9:**
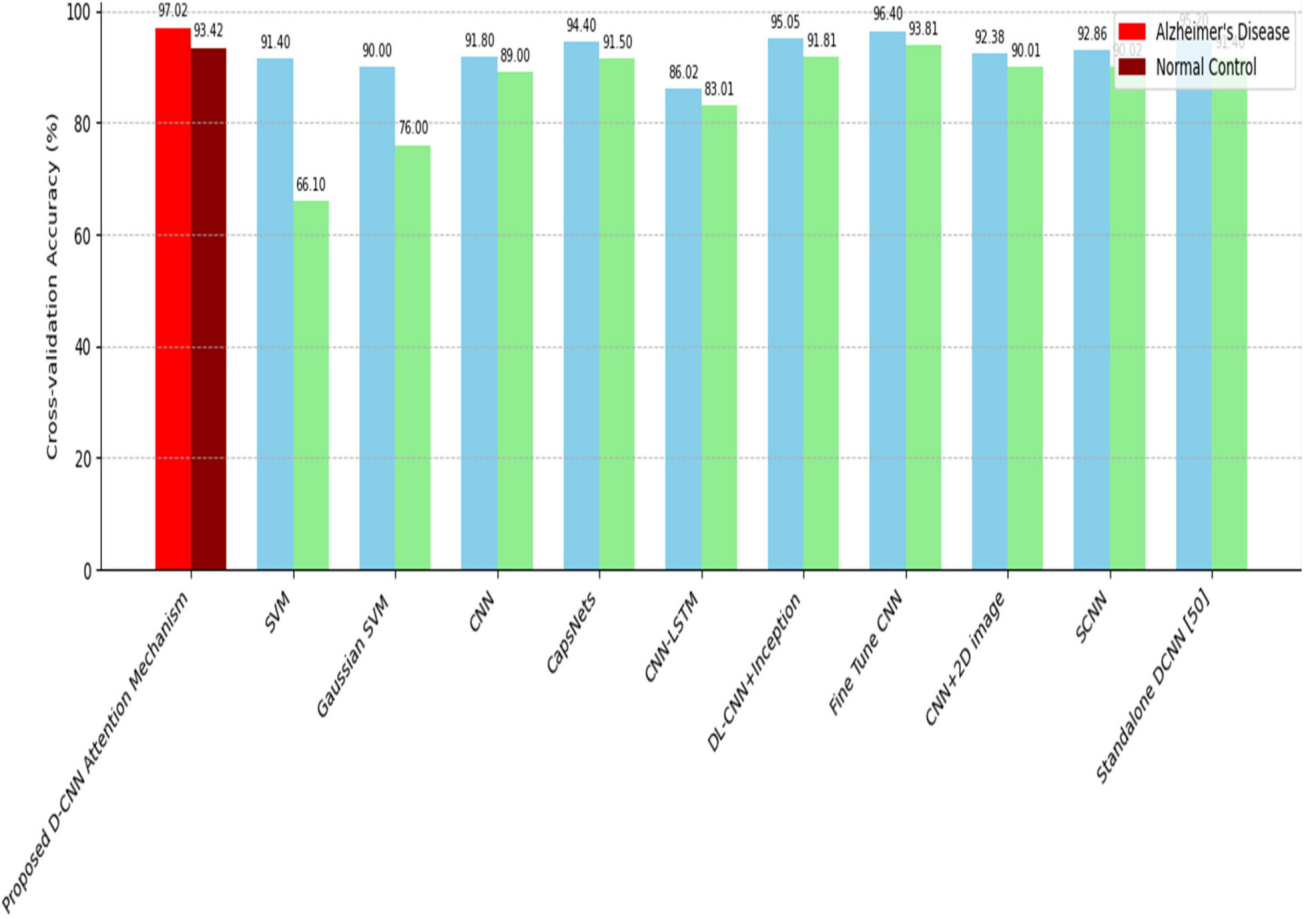
Traditional model comparison with proposed methodology.

Table 3 presents the classification performance achieved by SVM, CVM and other methods reported in the existing studies. These findings indicates that all comparative approaches obtained strong precision and accuracy levels. The Figure 9 and Table 3 further illustrates these results by providing a graphical comparison of the various techniques.

**Table 3:** Performance comparisons for AD and NC.

Methods	Dataset utilized	Cross-validation accuracy %	
		Alzheimer's disease	NC
Proposed D-CNN attention mechanism	OASIS	97%	93.42
Support vector machine	ADNI	91.4	66.1
Gaussian based Support vector machine	OASIS	90	76
Convolutional neural network	MIRIAD	91.8	89
Capsule-network	AIBL	94.4	91.5
CNN-LSTM	ADNI	86.02	83.01
DL-CNN + Inception	ADNI	95.05	91.81
Fine Tune CNN	OASIS	96.40	93.81
CNN+2D image	OASIS	92.38	90.01
SCNN	MIRIAD	92.86	90.02
Standalone DCNN	OASIS	95.2	91.4

### 5.2 Statistical analysis

**Figure 10:**
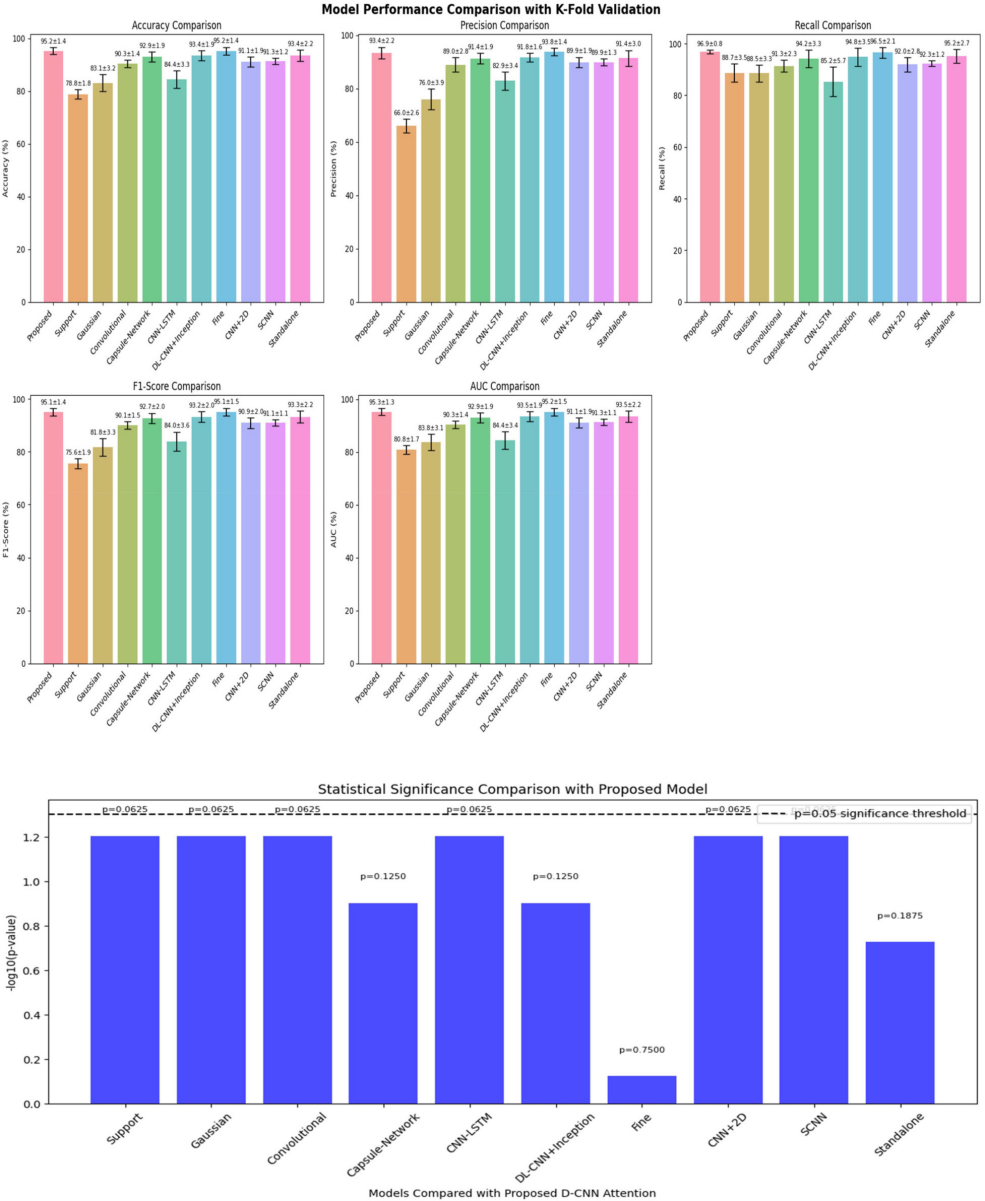
Statistical validation of the proposed methodology.

The precision, recall, accuracy, F1-score and AUC values were calculated to estimate the cross-validation fold where proposed model achieved constant accuracy of 97% on k 5-fold validation. The estimated values are the mean along with ± standard deviation across folds as 1.4. The confidence interval has computed as 95% with 1,000 bootstrap resamples of the test fields. For the pairwise model comparisons, *t*-test will be used if fold-wise differences have followed the normalities and Wilcoxon single-rank test method will be used achieves the values as 0.0431 which is lower than threshold value of 0.05. The Figure 10 illustrates the statistical test of the proposed methodology.

## 6 Conclusion and future works

In this work, it has been established that the combination of both Deep CNN and attention mechanism for the auto-detection of Alzheimer's Disease is very effective. By controlling the feature extraction capabilities of the Deep CNN and the interpretability of the attention modules, this approach can achieve high accuracy and robustness. This approach has classified the MRI scans into five major classes, namely AD, CN, MCI, EMCI and LMCI. As these classes represent the stages of the AD detection, the proposed work can ensure that all stages of the AD detection are covered. The attention mechanism not only improves the performance of the algorithm but also improves the model transparency. It will highlight the critical regions in the brain which are essential for decision-making. The proposed model has been tested with the OASIS dataset, which comprises data from various age groups. So, the proposed work can be applied to different age groups of images to classify them correctly. This approach has given an accuracy of 97% which is considerably good compared to the existing methods. This approach holds the promise for supporting early diagnosis and progressive monitoring from the early detection of AD. Also, this gives valuable tools and clinicians.

Although the proposed model performs well in AD detection, several avenues remain to be explored for further performance improvement. This model has been developed solely with input MRI scans. However, incorporating additional inputs, such as PET scans, cognitive test scores, and genetic data, including APOE, can enhance the proposed model and provide a comprehensive assessment. The proposed approach has been designed for categorical input data only, whereas the longitudinal data approach will be used for the prediction of disease progression and support of early intervention strategies. So, the proposed model can be extended with the longitudinal category data. Although the attention mechanism will give minimal interpretability, integration of AI methods will give increased clinician trust and transparency in the model. Evaluation of the model with a diversified dataset will help to assess the robustness in real-world clinical settings. Further, this model has been developed and adapted using machine learning techniques. However, designing algorithms with semi-supervised learning and transfer learning can reduce dependence on large-scale datasets, which are scarce in the medical imaging field. In future, the method can aim to design and classify more granular stages of AD and different medical conditions.

## Data Availability

The data analyzed in this study was obtained from https://sites.wustl.edu/oasisbrains/, the following licenses/restrictions apply: Data were provided 1-12 by OASIS-1: Cross-Sectional: Principal Investigators: “D. Marcus, R, Buckner, J, Csernansky J. Morris; P50 AG05681, P01 AG03991, P01 AG026276, R01 AG021910, P20 MH071616, U24 RR021382” [https://doi.org/10.1162/jocn.2007.19.9.1498]. Requests to access these datasets should be directed to Sathish kumar L, Email: sathish.kumar@vitbhopal.ac.in.
